# Impact of Paneth Cell Autophagy on Inflammatory Bowel Disease

**DOI:** 10.3389/fimmu.2018.00693

**Published:** 2018-04-05

**Authors:** Shu-Ling Wang, Bo-Zong Shao, Sheng-Bing Zhao, Jun Fang, Lun Gu, Chao-Yu Miao, Zhao-Shen Li, Yu Bai

**Affiliations:** ^1^Department of Gastroenterology, Changhai Hospital, Second Military Medical University and Naval Medical University, Shanghai, China; ^2^Department of Pharmocology, Second Military Medical University and Naval Medical University, Shanghai, China; ^3^Department of Gastroenterology, Zhongnan Hospital of Wuhan University, Wuhan, China

**Keywords:** autophagy, Paneth cell, inflammatory bowel disease, unfolded protein response, endoplasmic reticulum stress

## Abstract

Intestinal mucosal barrier, mainly consisting of the mucus layer and epithelium, functions in absorbing nutrition as well as prevention of the invasion of pathogenic microorganisms. Paneth cell, an important component of mucosal barrier, plays a vital role in maintaining the intestinal homeostasis by producing antimicrobial materials and controlling the host-commensal balance. Current evidence shows that the dysfunction of intestinal mucosal barrier, especially Paneth cell, participates in the onset and progression of inflammatory bowel disease (IBD). Autophagy, a cellular stress response, involves various physiological processes, such as secretion of proteins, production of antimicrobial peptides, and degradation of aberrant organelles or proteins. In the recent years, the roles of autophagy in the pathogenesis of IBD have been increasingly studied. Here in this review, we mainly focus on describing the roles of Paneth cell autophagy in IBD as well as several popular autophagy-related genetic variants in Penath cell and the related therapeutic strategies against IBD.

## Introduction

The intestinal tract functions in digesting food taken orally and absorbing nutrients from the materials in the gut lumen. Apart from this, the gut is also considered as a crucial immune organ due to the enormously diverse microorganism harbored in the gut. During this process, intestinal mucosal barrier plays a pivotal role in maintaining the peaceful coexistence with them, detecting, and eliminating the pathogenic microbial debris by triggering immune response and inflammatory reaction (1–5). In general, the intestinal defense system is composed of three parts, including the mucus layer, intestinal epithelial cells (IECs), as well as other cells related to the innate immune system (6). However, once the intestinal mucosal barriers are damaged or the microbial balance is disturbed, the immune and inflammatory responses will be over-activated, along with the accumulation of the reactive oxygen species (ROS) and disturbance of mitochondrion in function (7, 8). Those responses may contribute to the pathogenesis of inflammatory bowel disease (IBD) (9). Consequently, inhibiting the over-triggered inflammatory and defensive responses may serve as a potential and effective treatment for IBD. Among all of IECs, Paneth cells were reported to play a vital role in regulating the microbial composition, the innate and adaptive immune responses to the host, and the inflammatory reaction (10–12). Autophagy is a self-protecting response to various stresses, which plays a pivotal role in physiological processes, such as secreting proteins, producing antimicrobial peptides (AMPs) and degrading aberrant organelles or proteins, and thus easing the over-activated inflammation and self-defensive responses (13–15). Dysfunction of autophagy is regarded as a vital factor in the pathogenesis of IBD, which may be related to the impairment of the bacterial killing, antimicrobial materials secretion, and so on. Given those evidence, an increasing number of researches are focusing on the role of autophagy in developing a novel therapeutic strategy to fight against inflammation or immune-related diseases, including IBD. Here, in this review, we summarized the current understanding of IBD and autophagy and most importantly, the roles of Paneth cell autophagy in IBD as well as several autophagy-related genetic variants in Paneth cell and therapeutic strategies against IBD.

## Intestinal Defense System and IBD

### Intestinal Defense System

It is widely acknowledged that the intestinal tract is exposed to trillions of harmful antigens in food, factors derived from commensal and pathogenic microorganisms, as well as immune signals which is underneath the epithelium (1, 2, 16, 17). As a result, the intestinal defense system plays a significant role in maintaining the homeostasis between the host and microbial community. Generally speaking, various kinds of proteins, lipids, and carbohydrates accumulate in water, forming a gel-like layer on the surface of mucosa in the intestinal mucus layer (18, 19). The mucus layer underneath is composed of two layers: the outer and inner layers (6, 20). Among all of components in the mucus layer, the AMPs, such as defensins and cathelicidins, protect the intestinal tract against microbes (21, 22). When pathogens intrude, mucus layers work together with IECs to form a physical and chemical barrier, generating various inflammatory responses and immune reaction *via* various specific and unspecific mechanisms (23). Apart from the mucus layers, IECs also form a central part of the intestinal defense system which work as an interface between the quantitative microbial ecosystem in the intestinal lumen and the relatively sterile environment of the internal body (3–5). Specifically speaking, the epithelium mainly consists of six types of IECs, including goblet cells, enteroendocrine cells, absorptive enterocytes, tuft cells, micro-fold villus cells, and Paneth cells (24). Goblet cells mainly secrete a great amount of mucin to build up the mucus barrier, while the enteroendocrine cells help to produce various neuropeptides and restore the intestinal tissue (25, 26). As the most abundant cell type, absorptive enterocytes secret a series of cytokines and chemokines, which play a pivotal role in regulating the diversity of the commensal microorganisms and the immune responses of subjacent mucosal (27). Paneth cells, first described by an Austrian physiologist called Joseph Paneth, initially located at the bottom of small intestinal crypts, are the key cells in this review for discussion. Paneth cells secret granules containing various AMPs and peptides, such as defensins-like human lysozyme, defensin (HD)-5 and -6, lysozyme, regenerating islet-derived 3 gamma (RegIIIγ) and phospholipase A2 group IIA (sPLA2), as well as inflammatory cytokines, such as transforming tumor necrosis factor α (TNF-α), growth factor β1 (TGF-β1), and prostaglandin E2 (28–34). Previous studies demonstrated the crucial roles of Paneth cells in fighting against the invasion of pathogens, modulating the commensal microbiota, regulating the innate immunity, as well as impacting the functions of intestinal niche (7, 8, 31, 35–39). Those studies will be described and discussed in detail in the following part of the contents.

### Pathogenesis of IBD

Generally speaking, IBD is mainly composed of two types, namely Crohn’s disease (CD) and ulcerative colitis (UC). CD is remarkable for skipping and transmural inflammation in the distal small intestine and colon with lymphoid aggregation. In terms of UC, the inflammatory areas are continuously extending from the rectum to the whole colon and the inflammation mainly confines to the mucosa and are featured by a mixture of various inflammatory cells. Recent reports demonstrated that IBD affected nearly 1.5 million people in America and led to major morbidity, especially among young people (40, 41).

Although the precise etiology of IBD remains to be unclarified, increasing evidence suggests that genetic, environment, and interactions between intestinal barriers and commensal microbiota may converge to trigger the initiation and progression of IBD (42). Epidemiological data provides evidence for the role of gene in the development of IBD: 15% of patients with CD would have an IBD-affected family member, and the concordance of CD in monozygotic twins is up to 59% which is much higher than in the dizygotic twins (only 10%) (43). Genome-wide association studies (GWAS) have recognized over 200 IBD susceptibility loci, which will be discussed in the subsequent contents of the review (44, 45). Besides, accumulating studies implicated various pathways in the development of IBD, including the modulation of the intestinal microbiota, over-triggered inflammation, abnormal innate or adaptive immune reaction, and endoplasmic reticulum stress (ERS) (46–48). In addition, environmental factors also play an important role in the onset and development of IBD and smoking is considered as a crucial environmental risk for the development of CD (49). Another environmental factor contributing to IBD is air pollution (50). It was reported that ozone or nitrous oxides could intrude into intestinal tract through food and water, increasing the permeability of IECs (51). Besides, a clinical study conducted by Larsson et al. showed that the mucin 2 was deficient in the majority of the active UC patients, which was associated with the severity of IBD (52). In addition, it was also reported that dysfunction of the immune reaction contributed to the pathogenesis and progression of IBD through the dysregulation of the IFN-γ/STAT1 pathway as well as the imbalance of Treg and Th17 cells in IBD (53, 54). Among various intestinal defense systems, the dysfunction of Paneth cells may be a crucial factor attributing to IBD by reducing the production of the antibacterial peptides and changing the diversity and quantity of intestinal microbiota. Furthermore, microbial profiling studies also have demonstrated the important role of dysbiosis in IBD onset (55). Although no causative microbe has yet been identified, plenty of evidence has focused on the expansion of opportunistic pathogens (“pathobionts”), such as adherent-invasive *Escherichia coli* strains (AIEC) (56). Finally, some special drugs, such as antibiotics was also a potential factor in the pathogenesis of IBD because antibiotics could alter the intestinal microbiota (57).

## Autophagy and IBD

### Autophagy and Its Functions

Autophagy is a conserved lysosome-dependent catabolic process, degrading and recycling protein aggregates and damaged organelles (58). Basal autophagy occurs in nearly all kinds of cells to maintain the homeostasis of amino acid pool (59). Autophagy is generally classified into three types: macroautophagy, microautophagy, and chaperone-mediated autophagy (60, 61). During the process of microautophagy, lysosomal/vacuolar membranes invaginate so as to engulf intracellular components *via* a non-selective degradative mechanism (62). It was reported previously that chaperone-mediated autophagy could transport organelles and proteins into lysosomes only with the assistance of chaperones which were located in the lysosomal lumen (63). In the occurrence of macroautophagy, target materials, such as cytoplasmic components or invasive bacteria are surrounded by a double-membrane bound autophagosome. When autophagosome was combined with lysosome, it changes into a single-layer-membrane autolysosome with a strong degradative and digestive ability (64). Since macroautophagy is the most studied type, we will mainly explore the functions and roles of macroautophagy (hereafter referred to as “autophagy”) in IBD.

**Figure 1:**
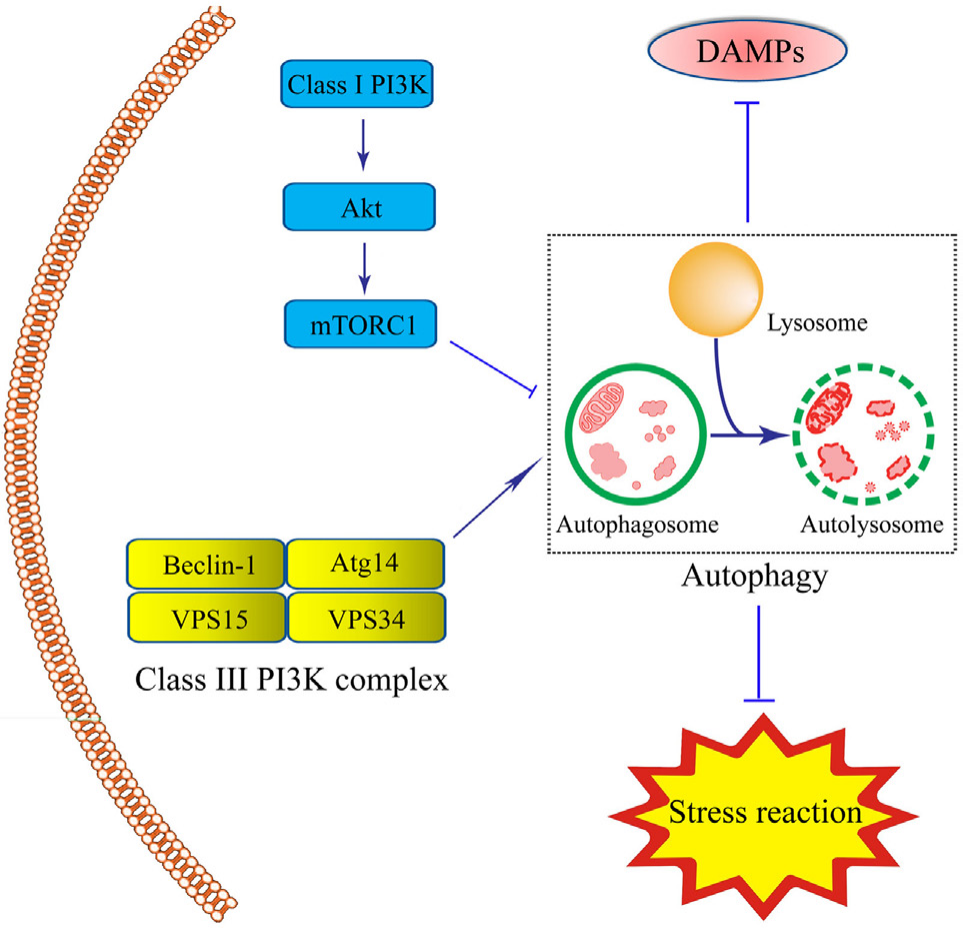
Schematic illustration of roles of intestinal epithelial cells (IEC) autophagy in the prevention of inflammatory bowel disease (IBD). Autophagy process in IECs is induced under inflammation- or immune-related challenge by the formation of the double-membrane autophagosomes. The integrity of autophagosomes and lysosomes leads to the formation of the single-membrane autolysosomes, functioning in degrading and recycling misfolded proteins, or dysfunctional organelles, thus contributing to cellular protection. The Class III-PI3K-Beclin-1 signaling pathway, led to the formation of Class III complex (Beclin-1-Atg14-vacuolar protein sorting(VPS)15-VPS34 complex) is the major inductive pathway to autophagy process, while the Class I-PI3K-mammalian target of rapamycin signaling pathway led to the inhibitory effects on autophagy process. The induction of autophagy protects IECs under stress through the degradation of various kinds of the damage-associated molecular pattern molecules and suppression of stress reaction.

There are two steps involved in the process of autophagy. In the first step, the cup-shaped double-membrane phagophores are shaped in the cytoplasm, and then engulf misfields proteins, damaged organelles or bacteria to become spherical double-membraned autophagosomes. Autophagosomes are usually considered to be produced from the nucleation and membrane expansion of phagophores. During the second step, autophagosomes fuse with lysosomes and endosomes to form the singer-lipid layer autolysosome, which is regarded as basal units for degradation and digestion (65). Autophagy process is induced by the detection of various specific cues, such as starvation or the invasion of microbes (66, 67). So far, two proteins are involved in the regulation of autophagy, including the mammalian target of rapamycin (mTOR) as an inhibitor and adenosine monophosphate-activated protein kinase as an inductor (65). The mTOR is often activated by lower levels of adenosine triphosphate (ATP) caused by nutrient sufficiency or several growth factors stimulation. It is triggered by the activation of Class I PI3K-mTOR *via* the phosphorylation of Akt pathway and formation of mTOR complex-1. This complex prevents the formation of autophagosome by inhibiting Atg1 (66, 68). The inductive signaling pathway is usually triggered when there is deficient nutrition, inflammation, or ROS stress. In this process, the Class III PI3K complex is formed by Beclin-1, Atg14, vacuolar protein sorting (VPS)15, and VPS34, leading to the assembly of the Atg12–Atg5–Atg16L complex and Atg8/LC3. Initiation of this signaling pathway plays a significant role in forming autophagosomes (Figure 1).

So far, increasing studies have demonstrated the crucial role of autophagy in maintaining cell and tissue homeostasis by regulating various physiological processes, including the clearance of pathogen, presentation of antigen, formation of cytokines, inflammatory responses, and the innate and adaptive immune reaction (61, 69, 70). Autophagy is widely regarded as a vital regulator in various kinds of diseases (71). Among all the diseases, the interaction between autophagy and IBD has been extensively explored and will be discussed in the following contents.

### Roles of Autophagy in IBD

As we mentioned above, IBD is a chronic and idiopathic inflammatory disease related to the over-triggering of inflammatory and immune responses in the gut (41). Autophagy affects the pathogenesis of IBD in multiple ways, including clearance of invading pathogens, secretion of antimicrobial materials from Paneth cells, presentation of antigen, and pro-inflammatory cytokine production by macrophages. One of the most important processes was to modulate the clearance of intestinal pathogenic microbes *via* the innate immune responses (72). When pathogens invade into the host cells, cytoplasmic vesicles in cellular plasma envelope them to form autophagosomes, thus confining them by absorbing nutrients and encouraging the acidification of micro-environment. The enhancement of autophagy promotes the combination between autophagosome and lysosome, contributing to the degradation of intracellular pathogens (also termed as xenophagy), such as *Listeria* and tuberculosis (73–75).

In addition, autophagy was reported to promote the survival of various cells, including IECs and neutrophils, through protecting from microbial toxins (74). It was reported that impaired autophagy could disturb the function of IECs and influence the inflammatory and immune responses, ROS production, and ERS, thus ultimately promoting the occurrence and development of IBD (76–79). Furthermore, it is noted that autophagy plays a vital role in the degradation of the damage-associated molecular pattern molecules (DAMPs), contributing to the alleviation of IBD. In general, DAMPs refer to various kinds of endogenous materials produced by stressed, impaired, dying or dead cells, covering the DNA, RNA, ATP, histones, hyaluronan, uric acid, heparin sulfate, the S100A calgranulins, interleukin (IL)-1 family members, heat shock proteins, and chromatin-associated high-mobility group box 1 (HMGB1) (80, 81). Previous studies demonstrated that the levels of DAMPs in serum fecal or mucosa of IBD patients or animal models were elevated significantly (82–84). Although autophagy was reported to enhance the degradation of DAMPs (85), it is worth mentioning that the induced autophagy in certain cases like starvation will greatly promote the release of DAMPs, such as ATP and HMGB1 (86, 87). As a result, to ultimately take advantage of the inhibitory effects of autophagy on DAMPs, further studies are demanded in this issue.

## Paneth Cell, IBD, and Autophagy

### Paneth Cells and IBD

As noted above, the mucus layer and IECs build up a physical and chemical barrier to prevent the invasion of pathogenic microbes, coexisting with commensal and beneficial microorganisms to maintain the homeostasis in the gut. In Paneth cells, they contain a great amount of secretary granules storing various AMPs, including HD-5, HD-6, lysozyme, RegIIIγ, and sPLA2, which largely influence the intestinal inflammatory and immune responses (11, 88–92). A recent study reported that Paneth cells were an original site for intestinal inflammation, such as IBD, which could regulate inflammatory reactions *via* the release of AMPs and other peptides, including IgA, lysozyme, phospholipase A2 and B, matrix metalloproteinase-7, lipopolysaccharide-binding protein, and several inflammatory cytokines (11, 93–95). AMPs are regarded as the most important antimicrobial substances in mucus layer by modulating the diversity and quantity of the intestinal microbiota and clear the invading pathogens (21). One of the most important AMPs is lysozyme, which is mainly produced by Paneth cells. The function of lysozyme is to fight against Gram-positive bacteria by catalyzing the hydrolysis of the β(1,4)-glycosidic linkages between *N*-acetylmuramic acid and *N*-acetylglucosamine in the polysaccharide component (28). Besides, the production of defensin, another vital AMP, would be triggered when pathological microbes stimulated toll-like receptors and intracellular sensors, such as NOD2 and NOD-like receptor (NLRs), and the mutation of NOD2 might increase the susceptibility to Crohn’s disease due to the lack of defensins (29, 30). Several studies on human tissue or animal models have revealed the reduced level of α-defensins in Paneth cells and the consequently decreased antimicrobial activity, which was regarded as key pathogenic factors of ileal CD (31–33, 96). RegIIIγ also played a key role in killing the Gram-positive bacteria by binding to cell wall peptidoglycans and loss of the antimicrobial RegIIIγ in mice contributed to spontaneous colitis (33). In addition, some researchers found that RegIIIα could alter the colonic microbiota by decreasing the level of ROS (97). Furthermore, PLA2 released from Paneth cells also has antibacterial activity, particularly against Gram-positive and Gram-negative pathogens by releasing arachidonic acid (34).

### Mechanism of Paneth Cell Autophagy in IBD Alleviation

As we discussed above, several mechanisms in Paneth cells contribute to the pathogenesis and progression of IBD. It has been reported that autophagy process in Paneth cells plays an important role in the alleviation of IBD through the regulation of several mechanisms related to IBD, such as ERS, ROS, and intestinal microbiota, which will be discussed in the following contents in detail.

## Endoplasmic Reticulum Stress

**Figure 2:**
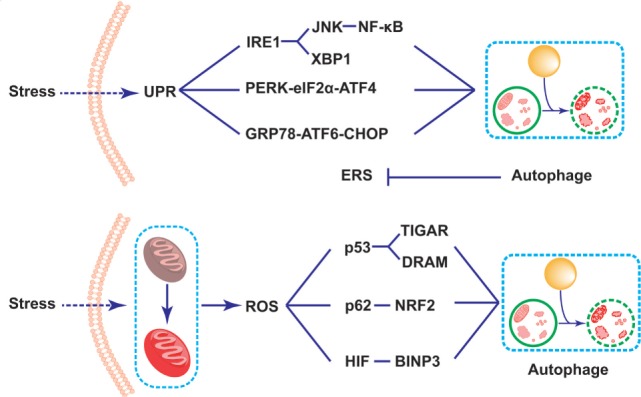
Schematic illustration of mechanisms of Paneth cell autophagy related to endoplasmic reticulum stress (ERS) and reactive oxygen species (ROS) in inflammatory bowel disease alleviation. Under the challenge of stresses, the triggering of the self-protected unfolded protein response process leads to the induction of ERS in Paneth cells. The ERS subsequently results in the induction of autophagy through three signaling pathways, including IRE1-JNK/NK-κB/XBP1, pancreatic ER kinase-eIF2d-activated transcription factor 4, and GRP78-activated transcription factor 6-CHOP signaling pathways. The induction of autophagy thus reduces the over-triggering of the ERS. In addition, the mitochondrial dysfunction triggers the accumulation of the reactive oxygen species (ROS) in Paneth cells. The triggering ROS largely induces autophagy process through the p53-TIGAR/damage-regulated autophagy modulator, p62-NF-E2-related factor 2, as well as BINP3 pathways, thus fighting against cellular damage under stresses.

It is well known that the unfolded protein response (UPR) plays an important role in the survival and functions of IECs in the production of proteins, which needs accurate management of endoplasmic reticulum (ER) (98, 99). The dysfunction of ER resulting from either genes or environmental factors causes abnormal UPR in the ER lumen, which is called ERS (35, 100). As Paneth cells are one type of secretory IECs which produce and release AMPs, they are particularly prone to ERS (98, 101, 102). Specifically speaking, ERS activates three kinds of protein residing in ER membrane to detect the UPR in ER lumen and resolve them: inositol-requiring transmembrane kinase endonuclease 1 (IRE1) *via* IRE1-JNk/nuclear factor-kappa B(NF-κB)/XBP1 signaling pathway, pancreatic ER kinase (PERK) *via* PERK-eIF2α-activated transcription factor (ATF)4 signaling pathway, and activated transcription factor 6 (ATF6) *via* GRP78-ATF6-CHOP signaling pathway (103–106). Selected gene deletion of one of these mediators in IECs will change the histological structure of the intestinal epithelium. For example, the XBP1-deleted IECs exhibit impaired Paneth cells, and thus leads to the dysbiosis and spontaneous intestinal inflammation mimic IBD, which may be probably induced by the activation of NK-κB pathway (89, 91, 94, 98). The second ERS-related IBD risk gene product, orosomucoid-like 3, is located in ER membrane, and takes part in protein folding and regulating UPR (107). Researchers argued that ERS-induced inflammation in Paneth cells possibly disturbed the microbial homeostasis, thus contributing to the pathogenesis and progression of IBD (98). Recent data showed that ERS could initiate the autophagy in Paneth cells *via* various pathways. Some argued that the induced process depended upon IRE1 by activating TRAF2 and ultimately JNK signaling (105, 108). Previous studies demonstrated that ERS could induce autophagy through the PERK-eIF2α-ATF4 pathway or IRE1-JNK pathway, which would ease NF-κB signaling pathway and relieve the ERS-induced inflammation in the intestine (108–111) (Figure 2). In addition, it was reported that dysfunctional autophagy in genomic manner significantly led to the over-triggering of ERS in experimental colitis animal model as well as IBD patients, thus largely exacerbating the severity of IBD (112, 113). For example, some researchers found that the number of Paneth cells in intestinal organoids lacing ATG16L1 was decreased, which might be related to the disruption of mitochondrial homeostasis (114). What is more, Bel et al. discovered an important role of secretory autophagy in maintaining host defense, and further showed the mechanisms how autophagy-related genes predisposed individuals to IBD. In this study, they found that ERS induced by the invasion of pathogens could trigger the secretory autophagy in Paneth cells, thus limiting bacterial dissemination (36). That study has been subsequently commented that secretory autophagy produced a vital effect on the secretion of lysozyme during bacterial infection of the gut (115, 116). Based on that evidence, taking advantage of autophagy process in the inhibition of ERS might serve as a potential therapy in the treatment of IBD.

Autophagy has been reported to inhibit the level of ERS in various inflammatory and immune diseases (117–119). In the pathogenesis of IBD, it was previously demonstrated that in experimental colitis mice model, the alleviative effects of TREM-1 on IBD severity was caused by the induction of autophagy and inhibition of ERS level (119). In addition, it was also demonstrated that dysfunction of autophagy resulted in the triggering of ERS in enterocytes, thus deteriorating the severity of IBD (118). A recent study showed that defective autophagy in IECs might predispose people to IBD *via* the decreased clearance of IRE1 during ERS (120). Those studies indicated the anti-ERS effect of autophagy in IBD.

## Reactive Oxygen Species

In addition, ROS is considered as conservational signaling molecules in nearly all cells, which plays a vital role in modulating cellular functions (121). Under normal conditions, ROS produces during the process of oxidative phosphorylation can be handled by intracellular antioxidants. However, once the production of ROS exceeds the generation of antioxidants, it may lead to various inflammatory disease including IBD (83, 122). As one of the most important source of ROS, dysfunctional mitochondria may modify the rearrangements of cytoskeleton, the framework of plasmalemma as well as the balance between kinases and phosphatases, thus promoting the internalization of microorganism and contributing to the onset of IBD (123). Furthermore, previous studies demonstrated the pivotal role of extracellular ROS in increasing the permeability of intestinal epithelial (124). For example, it was reported that mitoTEMPO, an antioxidant in mitochondrial, attenuated the severity of colitis induced by dextran sulfate sodium (DSS) through improving the function of the intestinal defense system (125).

The connection between autophagy and ROS has been increasingly studied recently in various kinds of diseases, including malignant tumors, neural disorders, metabolic diseases, as well as inflammation- and immune-related diseases, such as the colorectal cancer, chronic pancreatitis, and cardiologic disease (126–128). It has been demonstrated that the accumulation of ROS lead to the induction of autophagy (129). Although the specific mechanisms are not fully elucidated, the transcriptional regulatory mechanism is considered to be dominant (130). Generally speaking, several signaling pathways have been clarified. The first pathway is related to the increasing production of p53, which subsequently leads to the enhancement in transcription of two autophagy inducers, namely p53-induced glycolysis and apoptosis regulator (TIGAR) and DNA damage-regulated autophagy modulator (DRAM). In addition, there is another well-recognized pathway related to p62, an important autophagy-related protein, which combines with the increasingly produced NF-E2-related factor 2 induced by the accumulation of ROS, thus triggering the process of autophagy (131). Besides, several signaling pathways have been discussed in the previous studies, such as the hypoxia inducible factor-BCL2/adenovirus E1B interacting protein 3 (BINP3) pathways, and so on (132) (Figure 2).

In Paneth cells, the ROS-induced autophagy has been reported to facilitate in the treatment of IBD. Previous studies demonstrated that the ROS-mediated antibacterial autophagy (well-known as “xenophagy”) as well as the mitochondrial autophagy (well-known as “mitophagy”) in Paneth cells contributed greatly to the attenuation of IBD, thus probably serving as potential strategies for the treatment of IBD (37, 38). In addition, since autophagy was essential for maintaining the normal functions of mitochondria, several researches specifically showed that Atg mutations led to the elevation of ROS in several cells, including the Paneth cells *via* the dysfunctional mitochondria (7, 8, 37, 38). Furthermore, it should be noted that the absence of autophagy in Paneth cells largely enhanced the production of ROS and inflammatory cytokines, thus deteriorating the severity of IBD in DSS-induced colitis mice model (133). As a result, ERS may serve as a vital factor in the mechanism by which the abnormal autophagy in Paneth cells contributes to the onset or progression of IBD.

### Intestinal Microbiota

Trillions of bacteria, also called microbiota, colonize in the human intestinal lumen, which help the host to maintain healthy through multiple ways, including assisting the digestion and absorption, educating the immune system, regulating metabolism, and fighting against pathogenic microbes. Microbial imbalance (dysbiosis) contributes to a wide range of diseases, including metabolic syndromes, autism spectrum disorders, IBD, and so on. Among all of the various pathways in maintaining the microbial homeostasis, one of the most important pathways is xenophagy, which refers to a pathogen clearance regulated by autophagy (73). The impeded autophagy induced by bacterial plays a vital role in CD, indicating the common defected handling of the microbiota in the gut. For example, studies have shown the lower level of xenophagy led to characteristic alterations in intestinal microbiota in CD patients, including one specific strains of *E. coli*, namely AIEC, colonizing the intestinal epithelial (134, 135).

Burger et al. found that the microbiota could induce basal Paneth cell autophagy by IFN-γ so as to maintain intestinal homeostasis (39). However, as we mentioned above, impaired sensing and handling of intracellular microorganisms by IECs is a central part in pathogenesis and progression of IBD. Paneth cells, as one of the main producers of AMPs, were reported to play a key role in sensing and modifying the compositions of microbiota in the intestine (136). For example, the impaired Paneth cells in IBD patients produced lower levels of defensins and lysozymes, which reduced the antimicrobial ability to fight against quantitative bacteria in the intestinal lumen (31). It was reported that the dysfunction of Paneth cell autophagy disturbed the intestinal microbiota, leading to the higher level of AIEC and *Salmonella typhimurium* intracellular survival (55). Increasing studies showed that the dysfunctional Paneth cell autophagy which was caused by the mutation of autophagy-related genes could impair the localization of invaded pathogens, the recognition of bacterial, the activation of antimicrobial reactions, as well as the release of various AMPs (60, 137). Accumulating evidence suggested that impaired autophagy of Paneth cells not only altered the composition of intestinal bacteria, but also led to improper responses to the changed microbiota (3, 4). For example, the impeded xenophagy made intestinal epithelium become hypersensitive to the products of the microbes, thus making the process of bacterial mishandling self-replicating and the onset of IBD (102, 138, 139). Interestingly, some researchers found that the administration of probiotics alleviated the severity of colitis, which might be a potential effective treatment for IBD (140, 141).

## Autophagy-Related Genetic Variants of Paneth Cells and the Therapeutic Role in IBD

Autophagy in IECs, especially in Paneth cells, is a highly conventional process which plays a vital role in maintaining intestinal homeostasis by degrading and recycling intracellular materials or organelles (142, 143). Increasing studies have shown the importance of autophagy in the pathogenesis and progression of IBD, which has been reported to be closely associated with various genetic mutations. Interestingly, vitamin D receptor (*Vdr*), autophagy-related 16-like 1 (*Atg16l1*), and nucleotidebinding oligomerization domaincontaining protein 2 (*Nod2*), as the best representative IBD-related gene variants, converge to be involved in Paneth cell autophagy (76, 144–147). Since autophagy controls the production and quality of lysosome in the granules of Paneth cells, impaired autophagy may probably result in the decreased level of AMPs and dysbiosis, thus contributing to IBD onset (146). In the following contents, those three genetic mutations in GWAS, including *Vdr, Atg16l1*, and *Nod2* as well as other genetic mutations, will be discussed on the connection with IBD in detail (summarized in Table 1).

**Table 1:** Genetic variants related to autophagy in Paneth cells.

Genetic variants	Types of variants related to inflammatory bowel disease (IBD)	Mechanisms	Application in IBD	Reference
*Vdr*	Mutation	Sensing the invading bacterial, regulating the expression of Nod 2	Butyrate	(152, 160–163)
*Nod2*	Mutation	Producing of α-defensins, sensing bacterial, forming autophagosome, regulating inflammatory response	–	(172–176)
*Atg16l1*	Mutation	Sensing bacterial, releasing AMP, forming autophagosome	–	(102, 180, 181)
*Xbp1*	Mutation	Regulating ERS	–	(121)
*Irgm*	Mutation	Bacterial killing, vacuolar trafficking and acidification, regulating autophagy	–	(38, 186)
*Atg4*	Deletion	Regulating inflammatory responses	–	(187)
*Tcf4*	Deletion	Regulating the expression of defensins and cellular differentiation	–	(188)
*Lrrk2*	Deletion	Regulating the function of autolysosome	–	(189–192)
*Atg5*	Deletion	Regulating the function of autolysosome		(39)

*Vdr, vitamin D receptor;Nod2, nucleotidebinding oligomerization domaincontaining protein 2; Atg16l1, autophagy-related protein 16-like protein 1; Xbp1, X-box binding protein 1; Irgm, immunity-related GTPase M; Atg4, autophagy-related gene 4; Tcf4, transcription factor; Lrrk2, leucine-rich repeat serine/threonine-protein kinase 2; AMP, adenosine monophosphate; ERS, endoplasmic reticulum stress*.

### Vitamin D Receptor

Vitamin D receptor is one of the most important nuclear receptors which mediates various activities of 1,25-dihydroxyvitamin D3 (vitamin D3), the activated form of vitamin D. When VDR is combined with vitamin D3, retinoid X receptors will heterodimerize with and activate VDR. After binding to vitamin D-response element, the activated VDR will regulate the transcriptional levels of various target genes to maintain the calcium homeostasis in electrolyte and blood pressure (148). Recent studies showed that vitamin D3 might act as a kind of hormone to regulate the innate and adaptive immune responses, suggesting the crucial role of vitamin D3/VDR system in pathology of various diseases (149–151). For example, vitamin D prevented the invasion of *M. tuberculosis* in lungs, benefited the gut microbiota and improved glucose balance in diabetes. What is more, vitamin D3 pathway could regulate the process of autophagy, such as induction, elongation, engulfment, and maturation, indicating the possible role of VDR in IBD (152).

Low levels of vitamin D and VDR in expression has been shown in IBD patients (153–155). A North–South gradient in the incidence of CD indicated that vitamin D deficiency might contribute to the onset of IBD (155). In addition, patients with polymorphisms of VDR were much more prone to IBD and the same trend was also presented in experimental animal colitis model, where *Vdr* knockout mice developed spontaneous colitis (156–159). Although these findings suggest the close relationship between VDR signaling and IBD, the specific pathway is still unknown. Generally speaking, the target genes of *Vdr*, include genes for cathelicidin antimicrobial peptide/interleukin-37 (LL-37) (producing cathelicidin), defensin beta/b (DEFB/b, producing defensins), CLDN2 (encoding claudin 2), and ATG16L1 (related to autophagy), which are mainly responsible against pathogens and maintain the intestinal microbe homeostasis (160, 161). For example, it was reported that the pro-inflammatory NF-κB pathway and autophagy might played a key role in initiating colitis in mice without *Vdr* (162). Accumulating studies also indicated that *Vdr* deletion promoted colitis by activating the NF-κB pathway (162, 163). The deficiency of *Vdr* was reported to reduce the level of IκBα, an endogenous inhibitor of NF-κB activity, thus promoting the activation of NF-κB pathway, and leading to intestinal inflammatory responses (164). On the other hand, VDR and autophagy are all involved in the onset of IBD (165). Several studies have considered vitamin D as a possible stimulator of autophagy in the infection of *M. tuberculosis* or HIV infection (166, 167).

In addition, some researchers hypothesized that the deficient *Vdr* in IECs reduced the expression of ATG16L1, thus impairing the antimicrobial functions of Paneth cells and increased the bacterial loads in intestinal mucosa and subsequently contributing to the onset of IBD (163). Actually, VDR signaling is a critical factor which regulates nearly 3% of human genomes, indicating its fundamental roles in the pathogenesis and treatment of IBD (168). It was shown that the deletion of *Vdr* in IECs increased the susceptibility of colitis induced by DSS by altering the composition of intestinal microbiota, such as the decreased amount of Butyrivibrio (163). However, this dysbiosis was reported to be corrected by fecal transplantation (134). Consequently, there are several possible therapeutic strategies for IBD treatment related to VDR: (1) the administration of bacterial products, such as butyrate which has been shown to increase the expression of VDR and suppress inflammatory responses in a colitis animal model; (2) enhancing intestinal VDR expression which enhances the induction of AMPs to kill pathogenic microbes; and (3) fecal transplantation which helps to rebuild up the intestinal microbial homeostasis and fight against pathogens. However, all of those potential treatments need further explorations.

### *Nod2* 

Nucleotidebinding oligomerization domaincontaining protein 2 (NOD2), a member of the NLR family, was the first susceptibility gene which was closely associated with CD (30, 169). As an intracellular sensor of muramyl dipeptide (MDP), much attention has been paid attention to on NOD2 in macrophages (170), while accumulating evidence also showed the vital role of NOD2 in Paneth cells (30, 32, 46, 47, 169, 171). The first study in German population revealed an obvious decrease of Paneth cell defensins in patients with *Nod2* mutations (32). Further analysis in Cleveland Clinic (US) illustrated that this drop was associated with specific *Nod2* 3020insC frameshift mutation (SNP13) (31).

Several mechanisms may lie in this process, such as the impaired autophagy, decreased bacterial sensing, lower levels of αdefensin, and altered immune tolerance by suppressing TLR signals. One of those important mechanisms is the impact of NOD2 on autophagy. In normal cases, once the NOD2 was activated by MDP and pathogens, it could recruit ATG16L1 to sites of bacterial entry and induce autophagosomes in dendritic cells and epithelial cells (172, 173). For example, CD patients with *Nod2* variants were reported to lack this ability and caused decrease in the function of killing intracellular bacteria, such as AIEC, *S. typhimurium*, as well as *Shigella flexneri* (134, 135, 172, 173). A similar distinct decrease in AMPs was also observed in *Nod2* knockout mice (138). In addition, the transcellular permeability and bacterial translocation were also increased in *Nod2* knockout mice, which might be caused by the reduced production of defensins particularly in Paneth cells (174–176). Apart from the impairment of those functions in the innate immune, studies also reported that the dysfunction of *Nod2* might also led to an obvious reduction in the adaptive immunity by decreased bacterial handling in dendritic cells and impaired antigen presentation related to MHC class II on cellular surface (172).

Although its exact functions remain to be unclarified, NOD2 appears to play an important role in IBD through the activation NF-κB pathway and toll-like receptor pathways. When NOD2 was activated by MDP or bacteria in Paneth cells, it would induce autophagy *via* the NF-κB pathway (177). On the other hand, the autophagy was reported to promote the delivery of NOD2, thus enhancing the inflammatory responses (172). When facing a great demand of antimicrobial substance, ERS will be induced in Paneth cells, thus increasing the internalization of microbes and contributing to IBD onset (101). Increasing evidence demonstrated that the increased internalization of microbes was further amplified when the cells lack *Nod2*, which is mainly mediated by the ROS and MAPK pathways (47). Specifically speaking, when the epithelia were treated with DNP + *E. coli*, the level of IκB, an activation-related indicator of the NF-κB was decreased, suggesting the vital role of NF-κB signaling pathway intermediated by ROS (178). In addition, MAPKs-ERK1/2 pathway modifies paracellular and transcellular permeability of IECs to affect the microbial homeostasis in the gut (179).

### *Atg16l1* 

In 2007, the gene of autophagy-related protein 16-like protein 1 (*Atg16l1*), is one of the most important susceptibility genes, which was reported to be related to autophagy by GWAS (76). This was the first research to illustrate the interaction between autophagy and IBD, indicating that *Atg16l1* variants might lead to the dysfunction of Paneth cells. Actually, *Atg16l1* deficiency prevents the recruitment and combination of the ATG12-ATG5 complex, consequently impairing the engulfment of pathogens and cellular organelles during the process of autophagic catabolism (102, 180). Along with those lines, CD patients with ATG16L1 T300A risk loci exhibited disturbed capture of *S. typhimurium* in autophagosomes (181).

Recently, some studies provided strong evidence that Paneth cell autophagy imposed pivotal roles in the pathogenesis and progression of IBD (30, 32, 46–48, 102, 169, 182). In 2008, Cadwell et al. engineered an *Atg16l1*-deficient mouse and reported the defected Paneth cells which had fewer granules and decreased levels of antimicrobial substance inside (182). They also presented impaired exocytosis pathway of granules through which cellular components, including AMPs and other innate antibiotic peptides were transported to the intestinal lumen. Besides, the abnormal Paneth cell function would increase the production of inflammatory mediators, such as leptin and adiponectin, which were also elevated in IBD patients (183). Similarly, the morphologic changes and granule dysfunctions in Paneth cells was also observed in CD patients with *Atg16l1* mutation (102, 183, 184). Furthermore, Cadwell et al. additionally provided interesting data on this issue, showing that *Atg16l1* hypomorphic mice together with the infection of MNV CR6 caused abnormal granular secretion in Paneth cells, thus leading to intestinal lesions (48, 137). Recently, Matsuzawa-Ishimoto et al. found the mutation of ATG16L1 in intestinal led to the loss of Paneth cells, which was associated with the disturbance of mitochondrial homeostasis (114).

Taken together, those studies indicated that dysfunctional autophagy in Paneth cells induced by *Atg16l1* mutation might trigger the dysbiosis in intestine, which made individuals more susceptible to environmental to facilitate the onset of IBD.

### Others

Apart from those three common susceptibility genes for IBD, some other genes involving Paneth cell autophagy have been reported. Among those genes, some genes contributed to the pathogenesis and progression of IBD through genetic mutation. For example, genetic variant in *Xbp1* was reported to lead to the elevated ERS through the defection in UPR in highly secretory IECs, especially Paneth cells, thus impairing their functions and inducing the onset of IBD (98, 114). In addition, immunity-related GTPase M (*Irgm*) was also regarded as an IBD-susceptibility gene on chromosome 5q33.1 (185). IRGM is mainly associated with bacterial killing, vacuolar trafficking and acidification, and autophagy induced by microbes, thus maintaining the intestinal homeostasis. In 2008, McCarroll et al. reported a 20 kb deletion polymorphism upstream from Irgm which could suppress autophagy, thus impairing the ability to clear pathogens and contributing to the onset of IBD (186). In addition, a recent study showed that the location and granule of Paneth cells were greatly affected by IRGM, which was closely associated with the downregulated level of autophagy of Paneth cells (38). The induced Paneth cell autophagy would impair autophagic control of pathogens such as *S. typhimurium*, thus leading to dysbiosis and the onset of IBD (186).

Besides genetic mutation, several researches revealed that the deletion of some genes also affected Paneth cell autophagy. For example, a recent study focusing on *Atg4* genes considered Atg4B as a novel protective protein in regulating inflammatory responses during the pathogenesis of experimental colitis (187). They also found that the level of Atg4B was paralleled with the level of autophagy. Moreover, they found that the expression of Atg4B was obviously decreased in IBD patients, and Paneth cell in mice presented obvious abnormalities after deletion of Atg4B. They demonstrated that *Atg4b*-null mice model could be used to test new treatments for intestinal diseases associated with autophagy deficiency, including IBD. Those findings indicated the important role of *Atg4b* in Paneth cell autophagy and IBD. In addition, TCF was also shown to be associated with antimicrobial dysfunction of Paneth cells and the onset of CD. The deletion of *Tcf4* would cause a decreased production of AMPs in Paneth cells and impaired ability to fight against various pathogens by affecting the expression of α-defensin and the differentiation of cells (188). Recently, leucine-rich repeat serine/threonine-protein kinase 2 (*Lrrk2*), a CD-susceptibility gene, was reported to be correlated with xenophagy by affecting the degradation of autophagosome–lysosome (189). Studies demonstrated that the deletion of *Lrrk2* resulted in the lower level of lysozyme which might be caused by the impaired Paneth cells autophagy, thus contributing to the onset of IBD (190–192). What is more, a new study revealed the crucial role of the autophagy protein Atg5 in regulating the immune responses and protecting epithelial cells during acute intestinal inflammation. They reported that the specific deletion of Atg5 in Paneth cells contributed to the destruction of the crypts in structure, which was similar to changes in pan-epithelial *Atg5-*deficient mice. Additionally, lack of functional autophagy in Paneth cells led to impaired intestinal permeability. Thus, Atg5 expression in Paneth cells is crucial for tissue protection during acute gastrointestinal infection (39).

## Conclusion

In this review, we discussed the roles of Paneth cell autophagy in the pathogenesis and progression of IBD. We mainly focused on the popular mechanisms of Paneth cell autophagy in IBD alleviation, including the regulation of ERS, ROS, and intestinal microbiota. In addition, several well-studied genetic variants of Paneth cells and the related treatment roles in IBD were also summarized.

## Author Contributions

S-LW, B-ZS and S-BZ retrieved and analyzed concerned literatures. S-LW, B-ZS and S-BZ wrote the manuscript. JF and LG designed the table and figures. C-YM, Z-SL, and YB revised the manuscript. All the authors agreed to be accountable for the content of the work.

## Conflict of Interest Statement

The authors declare that the research was conducted in the absence of any commercial or financial relationship that could be constructed as a potential conflict of interest.
